# Targeting TFE3 Protects Against Lysosomal Malfunction-Induced Pyroptosis in Random Skin Flaps via ROS Elimination

**DOI:** 10.3389/fcell.2021.643996

**Published:** 2021-04-08

**Authors:** Jiafeng Li, Junsheng Lou, Gaoxiang Yu, Yijie Chen, Ruiheng Chen, Zhuliu Chen, Chenyu Wu, Jian Ding, Yu Xu, Jingtao Jiang, Huazi Xu, Xuwei Zhu, Weiyang Gao, Kailiang Zhou

**Affiliations:** ^1^Department of Orthopaedics, The Second Affiliated Hospital and Yuying Children’s Hospital of Wenzhou Medical University, Wenzhou, China; ^2^Zhejiang Provincial Key Laboratory of Orthopaedics, Wenzhou, China; ^3^Department of Obstetrics and Gynecology, The Second Affliated Hospital and Yuying Children’s Hospital of Wenzhou Medical University, Wenzhou, China; ^4^Department of Cardiovascular and Thoracic Surgery, The Second Affiliated Hospital and Yuying Children’s Hospital of Wenzhou Medical University, Wenzhou, China

**Keywords:** TFE3, pyroptosis, ROS, random skin flap, AMPK-MCOLN1 signaling pathway

## Abstract

Increasing evidence indicates that pyroptosis, a new type of programmed cell death, may participate in random flap necrosis and play an important role. ROS-induced lysosome malfunction is an important inducement of pyroptosis. Transcription factor E3 (TFE3) exerts a decisive effect in oxidative metabolism and lysosomal homeostasis. We explored the effect of pyroptosis in random flap necrosis and discussed the effect of TFE3 in modulating pyroptosis. Histological analysis via hematoxylin-eosin staining, immunohistochemistry, general evaluation of flaps, evaluation of tissue edema, and laser Doppler blood flow were employed to determine the survival of the skin flaps. Western blotting, immunofluorescence, and enzyme-linked immunosorbent assays were used to calculate the expressions of pyroptosis, oxidative stress, lysosome function, and the AMPK-MCOLN1 signaling pathway. In cell experiments, HUVEC cells were utilized to ensure the relationship between TFE3, reactive oxygen species (ROS)-induced lysosome malfunction and cell pyroptosis. Our results indicate that pyroptosis exists in the random skin flap model and oxygen and glucose deprivation/reperfusion cell model. In addition, NLRP3-mediated pyroptosis leads to necrosis of the flaps. Moreover, we also found that ischemic flaps can augment the accumulation of ROS, thereby inducing lysosomal malfunction and finally initiating pyroptosis. Meanwhile, we observed that TFE3 levels are interrelated with ROS levels, and overexpression and low expression of TFE3 levels can, respectively, inhibit and promote ROS-induced lysosomal dysfunction and pyroptosis during *in vivo* and *in vitro* experiments. In conclusion, we found the activation of TFE3 in random flaps is partially regulated by the AMPK-MCOLN1 signal pathway. Taken together, TFE3 is a key regulator of ROS-induced pyroptosis in random skin flaps, and TFE3 may be a promising therapeutic target for improving random flap survival.

## Introduction

Random-pattern skin flap grafting is conventionally used as a popular technique in reconstruction surgery, and it has been widely applied to repair and reconstruct massive deep skin damage and underlying soft tissue damages resulting from trauma and surgical procedures (Chang et al., 2017; Bai et al., 2018). However, the necrosis of flaps is one of the most common postoperative complications in the reconstructive surgery, which limits the length-to-width ratio of flaps to 1.5–2 (Milton, 1970; Li et al., 2019). Enhancing the vitality of random skin flaps and inhibiting necrosis in the distal region has important practical significance for improving the scope of the clinical application of random flaps.

The necrosis of random flaps is the result of a series of pathophysiological effects. The blood supply of the flap is mainly from the vascular network on the pedicle of the flap, and angiogenesis starts from the pedicle of the flap toward the distal end (Zhou et al., 2019). When the surgeon transects the vascular pedicle, the flap begins to undergo ischemic injury (Siemionow and Arslan, 2004). After neovascularization, ischemia-reperfusion injury (IRI) occurs in the random flap (Siemionow and Arslan, 2004). Restoration and reperfusion of the blood supply generates a large amount of reactive oxygen species (ROS) and neutrophils, which release a host of inflammatory factors such as interleukins to induce inflammatory cascades (Kim et al., 2012; Wu M. et al., 2018). Inflammatory cascades and ROS-induced oxidative stress further induce a cytokine storm, resulting in severe secondary injury (Ornellas et al., 2017; Wu M. et al., 2018). Finally, prolonged ischemia-reperfusion injury triggers cell death, caused by damage to cellular structures (Lopez-Neblina et al., 2005; Zhu et al., 2016). Therefore, alleviating tissue inflammation and oxidative stress and dampening cell death are considered to be crucial strategies for the prevention and treatment of the remote ischemic necrosis of random flaps.

Pyroptosis is a type of programmed cell death with the features of inflammation and cell death, which is newly identified. Different from apoptosis and necrosis, pyroptosis is characterized by caspase-1 activation, cell swelling, and membrane rupture, leading to leakage of proinflammatory contents and cytokines (Wu et al., 2019; Vinken, 2020). A variety of signaling pathways have been found to mediate the occurrence of pyroptosis, among which, NLRP3-caspase-1-GSDMD is the most classic (He et al., 2015). Nucleotide-binding oligomerization segment-like receptor family 3 (NLRP3), adaptor molecule ASC, and pro-caspase-1 constitute an inflammasome to activate caspase-1 together (Man and Kanneganti, 2016), which can activate pro-IL-1β and pro-IL-18 and cleave gasdermin D (GSDMD), forming macropores in the cell plasma membrane, thus triggering pyroptosis (Wang Y. et al., 2019). Studies have shown that NLRP3-mediated pyroptosis is an important pathological event that participating in the occurrence and development of many diseases, such as cardiovascular diseases and liver diseases (Wan et al., 2020). Random flap avascular necrosis is characterized by vascular disease. Thus, it is likely that pyroptosis may be involved in the ischemic necrosis of random skin flaps. However, it remains unclear whether the necrosis in the flaps is associated with pyroptosis.

Nucleotide-binding oligomerization segment-like receptor family 3 inflammasome assembly, is the critical step of pyroptosis, which is mediated by many inducements, such as ROS production, lysosome permeabilization (Derangère et al., 2014). Specifically, when lysosomal dysfunction occurs, increased membrane permeability causes cathepsin B to be activated and released into the cytoplasm, where it binds and activates with NLRP3, inducing pyroptosis (Jia et al., 2019). ROS disrupts the stability of lysosomes by oxidizing unsaturated fatty acids in the lysosomal membrane (Chen et al., 2012). Besides, ROS has been extensively studied as an important participant in ischemic flap necrosis (Suzuki et al., 1989). Therefore, we hypothesized that with the exhaustion of the antioxidant system’s scavenging capacity, excessive ROS gradually accumulated in the flap may cause lysosomal dysfunction, which in turn may lead to pyroptosis.

Increasing studies have found that TFE3 mainly plays a biological role in promoting the production of antioxidant enzymes and enhancing the activity of lysosomes (Martina and Puertollano, 2016). Under cellular stress, TFE3 binds to the CLEAR (coordinated lysosomal expression and regulation) element on a series of lysosomal genes (such as CTSD and Lamp2) to promote the biological activity of lysosomes (Perera et al., 2015). In addition, TFE3 can decrease intracellular ROS via promoting the expression of antioxidant genes (Lu et al., 2017). Consequently, these transcription factors may underlie the pathophysiology of the necrosis of flaps and thus may be used for therapeutic benefits. Our findings revealed that higher levels of nuclear translocation of TFE3 were detected in the distal flap (area II) compared to the proximal flap (area I) accompanied with drastic ROS accumulation and lysosomal malfunction. Hence, we speculate that TFE3 inhibits the level of oxidative stress and enhances lysosomal activity to inhibit cell pyroptosis, thereby promoting the survival of random skin flaps. It has been reported that the activation of AMPK- MCOLN1 channel, which induce TFE3 into the nucleus to exert bio-activities (Fernandez-Mosquera et al., 2019). Therefore, it is essential to confirm whether TFE3 is regulated by the signaling pathway in random skin flaps.

In the present research, we postulate that in flaps, excessive ROS accumulation leads to lysosomal malfunction and then triggers pyroptosis, which is harmful to the survival of the random skin flaps. Additionally, we hypothesize that TFE3 alleviates ROS-induced lysosomal malfunction and pyroptosis and improves the survival of random skin flaps. Utilization of pharmacological inhibitors, transgenic mice and short hairpin RNA, along with *in vivo* models, we found that in random skin flaps, the excess production of ROS leads to lysosomal malfunction and subsequently triggers pyroptosis, which was detrimental to flap survival. We revealed that TFE3 relieved ROS-induced lysosomal malfunction and pyroptosis. Finally, we found that the activation of TFE3 via the AMPK-MCOLN1 signaling pathway in the flaps.

## Materials and Methods

### Reagents and Antibodies

The reagents and antibodies applied in the research were as follows: Solarbio Science & Technology (Beijing, China): H&E Staining Kit, pentobarbital sodium, and diaminobenzidine (DAB) developer; Boster Biological Technology (Wuhan, China): Primary antibodies directed against Cadherin-5; Biogot Technology (Shanghai, China): GAPDH primary antibody; Protein Tech Group (Chicago, IL, United States): Primary antibodies against VEGF, GSDMD-*N*, histone-H3, and Cathepsin L; Cell Signaling Technology (Beverly, MA, United States): Primary antibodies against AMPK, p-AMPK and NLRP3; Abcam (Cambridge, United Kingdom): Primary antibodies directed against ASC, LAMP2, Caspase-1, Cathepsin D (CTSD), Cathepsin B (CTSB), TRPML1/MG-2, calcineurin A and CD34; Sigma-Aldrich Chemical Company (Milwaukee, WI, United States): Tiron (purity ≥98%), TFE3 and P-TFE3 primary antibodies; Abcolonal Technology (Wuhan, China): Primary antibodies directed against IL-18 and IL-1β; Boyun Biotechnology (Nanjing, China): Fluorescein isothiocyanate (FITC)-conjugated IgG secondary antibody, the ELISA kits of 8-OHdG, AOPP, and MDA; Santa Cruz Biotechnology (Dallas, TX, United States): 4,6- Horseradish peroxidase (HRP)-conjugated IgG secondary antibody; PerkinElmer Life Sciences (Waltham, MA, United States): The ECL Plus Reagent Kit; Thermo Fisher Scientific (Rockford, IL, United States): The NEPER nuclear and cytoplasmic extraction reagents, and the lysosome enrichment kit; Corning (United States): Transwell assays (8 μm); BD Biosciences (United States): Matrigel; Gibco (Grand Island, NY, United States): DMEM (glucose-free), fetal bovine serum (FBS) and 0.25% trypsin-ethylenediaminetetraacetic acid (trypsin-EDTA); the GeneChem Chemical Technology Co., Ltd., (Shanghai, China): The AAV-TFE3 shRNA adeno-associated virus (serotype 9), and the AAV-AMPK shRNA adeno-associated virus; MedChemExpress (Shanghai, China): MCC950 (purity ≥98%); Yeasen Biotech (Shanghai, China): Dihydroethidium probe kit.

### Production of TFE3-KI/wt Mice

We followed the methods of our previous research (Zhou et al., 2020). TFE3-KI/wt mice were generated by the Nanjing Biomedical Research Institute of Nanjing University. Transcript Tfe3-201 (ENSMUST00000077680.9) was selected to produce TFE3-KI/wt mice. The Tfe3-201 gene has 10 exons, with the ATG start codon in exon 1 and TGA stop codon in exon 10. H11-CAGTfe3-flag-polyA knock-in mice were produced via the CRISPR/Cas9 system. Cas9 mRNA, sgRNA, and donor were coinjected into the fertilized egg. sgRNA guides Cas9 endonuclease to cleave at the H11 site to form a double-strand break (DSB). Such breaks will be repaired and lead to CAG-Tfe3-flag-polyA inserted in the H11 locus. The pups were genotyped by PCR, and then, sequence analysis and southern blot analysis were performed.

### Animals

C57BL/6 mice (male, 20–30 g) were provided by the Laboratory Animal Center of Wenzhou Medical University (License No. SCXK [ZJ] 2015-0001). Surgery, treatments, and postoperative care strictly abide by the Guide for the Care and Use of Laboratory Animals of the China National Institutes of Health. All procedures were approved by the Wenzhou Medical University’s Animal Research Committee (wydw 2017-096). Mice were individually housed in standard experimental conditions, with a light/dark cycle of 12 h, and regular food and water were freely available before any experimental operation. Flap tissues of area II and area I of eighteen mice were used for the area II group and area I group, and these mice were not subjected to any drug treatment. Ninety mice were randomly divided into the control, MCC950, Tiron, control + scramble control, and control + AMPK shRNA groups. TFE3-wt/wt mice were used for the WT (wild type) group. TFE3-KI/wt mice were randomly divided into the TFE3 KI group, TFE3 KI + scramble control group, and TFE3 KI + TFE3 shRNA group.

### Random Skin Flap Model

After anesthesia with 1% pentobarbital sodium, a dorsal random flap model (1.5 cm × 4.5 cm) was built under the central fascia of the dorsal mouse, as described above (Lee et al., 2017; Li et al., 2020). Any the known blood supply artery of the flap was completely excised. Then, flaps were sutured to original position via 4–0 Mousse threads. The skin flap was averagely separated into three regions: the proximal (area I), intermediate (area II), and distal (area III) zones. All animals were sacrificed with excess pentobarbital sodium on Day 7 after injection, and the flap tissue was immediately removed for subsequent experiments after euthanasia.

### Drugs and AAV Vector Administration

The MCC950 and Tiron groups received daily intraperitoneal injections of 10 mg/kg MCC950 and 500 mg/kg Tiron starting from 7 days before surgery until they were euthanized; mice in the control group, control + scramble control group, and control + AMPK shRNA group were administrated with the same dose and course of normal saline. 14 days before the building of the animal model, the TFE3 KI + scramble control, TFE3 KI + TFE3 shRNA, control + scramble control, and control + AMPK shRNA groups were injected with 100 μl of intravenous viral vectors in PBS with 1 × 10^10^ packaged genomic particle into the tail vein. The mice of the WT group, TFE3 KI group and area II and Area I groups were not subjected to any drug treatment.

### General Flap Evaluation

We followed the previously described methods (Li et al., 2020). In briefly, the macroscopic development, and features of appearance and color of the flap were observed 7 days after the operation. Image-pro Plus imaging software (Ver. 6.0) was used to calculate the survivable area and determine the percentage of survivable area as follows: the extent of viable area × 100%/total area.

### Evaluation of Tissue Edema

This was performed as the previously described (Lin et al., 2018). On the 7th day after operation, samples of flaps were obtained from each group and weighed. Then, they were dehydrated in an autoclave at 50°C and weighed until the weight remained stable for at least 2 days. The moisture content was determined as follows: ([wet weight – dry weight]/wet weight)100%.

### Laser Doppler Blood Flow Measurement

The vascular network in the whole area of the random flap were measured by Laser Doppler Blood Flow (LDBF) (Abraham et al., 2016). On POD 7, the mice in each group were anesthetized and scanned using a laser Doppler instrument (Moor Instruments, Axminster, United Kingdom) that shows minute vessels under the skin. The protocol for LDBF measurements was depicted earlier (Zhou et al., 2019). The intensity of the blood flow was assessed by perfusion units (PU) and was calculated by Moor LDI Review software (Ver. 6.1).

### Hematoxylin and Eosin Staining

Specimens were stained by hematoxylin and eosin (H&E) staining kit as described (Li et al., 2020). The extracted specimens were fixed in 4% paraformaldehyde for 24 h, embedded in paraffin, and cut into 4-mm thick sections. Then, a light microscope (×200 magnification) was used to observe the slices to estimate histological changes such as edema, inflammatory infiltration, and microvascular regeneration.

### Immunohistochemistry

Immunohistochemistry (IHC) was performed as previously described (Li et al., 2020). The sections were deparaffinized with xylene and rehydrated using a graded ethanol. Sections were washed and then blocked with 3% H_2_O_2_. After antigen repair, the sample was sealed with 10% bovine serum albumin phosphate buffered saline. Then, the sections were incubated with antibodies at 4°C overnight against CD34 (1:100) and VEGF (1:200). Next, sections were incubated with HRP-conjugated secondary antibody. The stained samples were imaged under at 200× magnification through the DP2-TWAN image acquisition system (Olympus Corp., Tokyo, Japan). Images were calculated via Image-Pro Plus software (Ver. 6.0).

### Cell Culture and Grouping

The human umbilical vein endothelial cells (HUVECs) were obtained from the American Type Culture Collection (Manassas, VA, United States) and cultured in RPMI 1640 medium containing 10% FBS and 1% antibiotics (penicillin, 100 IU/ml; streptomycin, 100 μg/ml) under an atmosphere containing 5% CO_2_ at 37°C in a constant temperature incubator. HUVECs were divided into four groups: normal control group; Oxygen and Glucose Deprivation/Reperfusion (OGD/R) group; OGD/R + Con-siRNA group; and OGD/R + TFE3-siRNA group.

### Oxygen and Glucose Deprivation/Reperfusion Model

We built the OGD/R model in HUVECs to simulate ischemia reperfusion injury of flaps. HUVECs were nurtured in glucose-free and serum-free DMEM medium in a hypoxia chamber (Thermo Scientific, United States) involving a gaseous mixture of 5% CO_2_ and 95% N_2_. After hypoxia for 6 h, the medium was replaced with RPMI 1,640 medium containing 10% FBS. Then, transferd HUVECs to the previous normal oxygen culture environment for 6 h. For migration and angiogenesis, the culture medium cannot be changed due to the experimental operation, and we only divised a 4-h glucose oxygen deprivation (OGD) to better estimate cell.

### TFE3-siRNA Cell Transfection

A short interfering RNA (siRNA) targeting the human TFE3 gene was designed and synthesized. The sequence is as follows: *GCTACACTCTGCATCGT* (RiboBio, Guangzhou, China). The cells were seeded in six-well plates and cultured to 60–70% confluence. Cells were then transfected with TFE3-siRNA or negative control using a Lipofectamine 2000 siRNA transfection reagent for 36 h (Thermo Fisher, Logan, UT, United States) according to the manufacturer’s instructions. After that, the cells suffered OGD or OGD/R injury, then were used for detections immediately.

### Enzyme-Linked Immunosorbent Assay

Enzyme-Linked Immunosorbent Assay (ELISA) was performed as previously described (Zhou et al., 2020). At a word, the flap tissue and cell supernatant were homogenized with PBS, and then, they were repeatedly frozen and thawed with liquid nitrogen. Then, they were centrifuged at 10,000 *g* for 10 min under 4°C conditions, and the supernatant was collected for ELISA detection. An ELISA kit was used to detect the levels of MDA,8-OHdG, and AOPP in tissue according to the manufacturer’s instructions (Boyun Bio, Shanghai, China).

### Western Blotting

After extracting the total protein from the flap tissues and cells with RIPA lysis buffer, we used the BCA assay to calculate the protein content. Cytoplasmic and nuclear protein were abstracted by NE-PER. Lysosomal and cytoplasmic protein were separated by the lysosome enrichment kit. An equal amount of 60 μg of tissue protein (30 μg of cellular protein) was separated by 12% sodium dodecylsulfate-polyacrylamide gel electrophoresis, transferred to a polyvinylidene difluoride membrane, and blocked with 5% non-fat milk. The membranes were incubated with the primary antibody against IL-1β (1:1,000), GSDMD-N (1:1,000), caspase-1 (1:1,000), VEGF (1:1,000), IL-18 (1:1,000), cadherin-5 (1:1,000), CTSD (1:1,000), GAPDH (1:1,000), AMPK (1:1000), p-AMPK (1:1000), ASC (1:1,000), TFE3 (1:1000), P-TFE3 (1:1000), CTSL (1:1000), LAMP2 (1:1000), NLRP3 (1:1,000), TRPML1/MG-2 (1:1000), calcineurin A (1:1000) and histone-H3 (1:1000) overnight at 4°C. Then washing with TBST, membranes were incubated with respective secondary antibody. The blots were visualized via the ECL Plus Reagent Kit. Quantification of the blot was conducted by Image Lab 3.0 software (Bio-Rad, Hercules, CA, United States).

### Immunofluorescence Staining (IF)

HUVECs were cultured on 12-well culture plates. After all stimulation, the medium was removed, and PBS was used before fixing with 4% paraformaldehyde. Next, the cells were permeabilized with 0.5% Triton X-100. Then, cells were sealed with bovine serum albumin incubated with primary antibodies against caspase-1 (1:200) and TFE3 (1:200) overnight at 4°C. For tissue sections, procedures before primary antibody incubation are the same as described in IHC, and then, sections were incubated with primary antibodies against caspase-1 (1:200), TFE3 (1:200), and LAMP 2 (1:200) overnight at 4°C. The second day, all sections and plates were washed, incubated for 1 h with FITC-conjugated secondary antibody and stained with DAPI. All images were evaluated under a fluorescence microscope (Olympus, Tokyo, Japan).

### Reactive Oxygen Species Measurement

The ROS levels of cells and tissues were detected by the DHE (Dihydroethidium) assay kit as previously described (Liu et al., 2020). All images were evaluated under a fluorescence microscope.

### Cells Migration and Tube Formation

These assays were performed as previously described (Wang et al., 2018). Transwell assays were performed to estimate the migration ability of the HUVECs. Cells were placed into the upper chamber, while the lower chamber involved glucose-free DMEM culture medium with 1% FBS, and then, the cells were placed in a hypoxia chamber. Cells treated with RPMI 1640 medium with 1% FBS and placed in a normoxic incubator were used as a normal control. After incubation, the upper cavity membrane was removed, followed by fixing with 4% paraformaldehyde, staining with crystal violet, and measurement of the migrated cells. The migrated cells were calculated using an inverted microscope (Nikon, Japan). A tube formation assay on Matrigel (BD Biosciences, United States) was used to estimate the HUVEC morphogenesis and tube formation capacity. Briefly, Matrigel solution was thawed at 4°C overnight and placed in a μ-Slide in a cell incubator to solidify. Cells are transfected with TFE3-siRNA or Con-siRNA, which were treated with serum-free and glucose-free DMEM culture medium, were seeded onto the Matrigel surface and placed in hypoxia chamber. Cells treated with RPMI 1,640 medium with 10% FBS and placed in a normoxic incubator were used as the normal control group. The migrated cells were calculated using an inverted microscope.

### Statistical Analysis

Statistical analyses were performed using SPSS statistical software program 24.0. The data are presented as the mean ± SEM. The *t*-test was applied for the comparison between two independent groups. When more than two groups were compared, one-way analysis of Variance (ANOVA) was used to statistically evaluate the data and Bonferroni correction was used for *post hoc* comparisons. *P*-values < 0.05 were regarded as statistically significant.

## Results

### NLRP3-Medicated Pyroptosis Exists in the Random Skin Flap Model and OGD/R Cell Model

**FIGURE 1:**
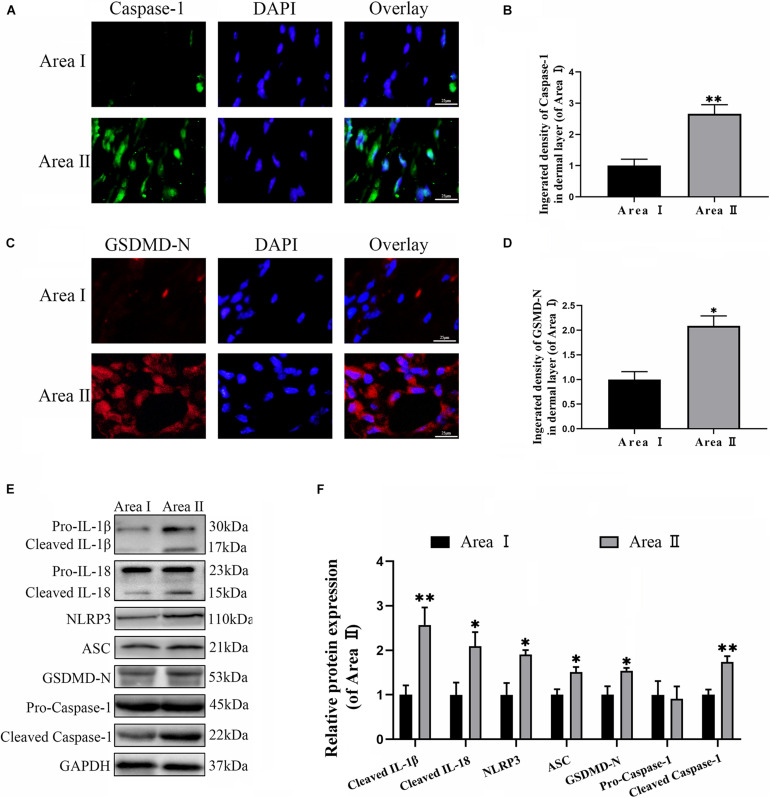
Cell pyroptosis exists in the random skin flap. **(A)** The levels caspase-1 of the flaps in the area II and area I groups evaluated by immunofluorescence (IF) (scale bar: 25 mm). **(B)** The fluorescence intensity for caspase-1. **(C)** Immunofluorescence for Gasdermin D–N-terminal domain (GSDMD-N) in skin flaps (scale bar: 25 mm). **(D)** The fluorescence intensity for GSDMD-N. **(E)** Western blotting of pyroptosis related proteins in flap tissue from area II and area I groups. **(F)** Optical density analysis of pyroptosis related proteins expression. Values represent the mean ± SEM, *n* = 6 per group. *p < 0.05 and **p < 0.01, vs. area I group.

To explore the level of pyroptosis in the flaps, the related markers were detected using IF and western blotting. As expected, the frequency of GSDMD-N and caspase-1 positive cells were greater in the intermediate flap (area II) compared to in the proximal flap (area I) (Figures 1A–D). Furthermore, the results from western blotting revealed that higher levels of the pyroptosis related markers (NLRP3, ASC, GSDMD-N, Caspase-1, IL-18, and IL-1β) were detected in the intermediate flap (area II) (Figures 1E,F). The above animal experiments found that there was enhanced pyroptosis in the distal part of the ischemic flap. To verify these results, we studied the vascular endothelial cell OGD/R model to simulate ischemic reperfusion. We further found that pyroptosis-related markers of (NLRP3, ASC, GSDMD-N, Caspase-1, IL-18, and IL-1β) gradually increased with the duration of hypoxia, arrived a peak in 6 h and subsequently remained stable in cells (Figures 7A,B). These findings indicated that pyroptosis exists in the ischemic flaps.

### NLRP3-Mediated Pyroptosis Contributes to the Necrosis of Flaps

**FIGURE 2:**
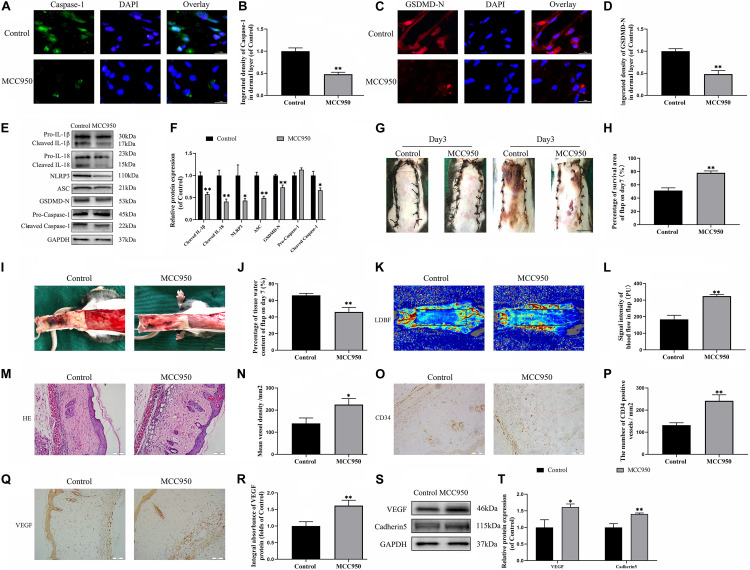
NLRP3-mediated pyroptosis triggers the necrosis of flaps. **(A)** Immunofluorescence to evaluate the caspase-1 level was conducted to exhibit the pyroptosis level in the skin flaps (scale bar: 25 mm). **(B)** The fluorescence intensity of caspase-1 analyzed using ImageJ. **(C)** Image of the immunofluorescence staining of GSDMD-N in skin flaps (scale bar: 25 mm). **(D)** The fluorescence intensity for GSDMD-N. **(E)** Western blotting of pyroptosis related proteins in flap tissue from the control and MCC950 groups. **(F)** Quantification of pyroptosis related proteins immunoblots. **(G)** Digital images of flap necrosis from the control and MCC950 groups (scale bar, 1.0 cm). **(H)** Histogram showing the percentage of viable flap area. **(I)** Digital images of the inner side of the random flap in each group exhibiting tissue edema (scale bar, 1.0 cm). **(J)** Quantification of the flap tissue water content. **(K)** Full field LDBF images of flaps by LDBF (scale bar, 1.0 cm). **(L)** Quantification of signal intensity of blood flow. **(M)** Morphologic observation of flap tissue by H&E staining (original magnification, ×200; scale bar, 50 μm). **(N)** H&E staining to show vessels. **(O)** The expression of CD34 to mark vessels was evaluated by immunohistochemistry staining (original magnification, ×200; scale bar, 50 μm). **(P)** Histogram showing the CD34-positive vessel densities **(Q)** Immunohistochemistry for VEGF on the skin flaps from the control and the MCC950 groups (original magnification, ×200; scale bar, 50 μm). **(R)** Histograms of optical density values for VEGF in IHC. **(S)** Western blotting of VEGF and cadherin-5 in flap tissue from the control and MCC950 groups. **(T)** Quantification of VEGF and cadherin-5 immunoblots. Values represent the mean ± SEM, *n* = 6 per group. *p < 0.05 and **p < 0.01, vs. control group.

To further confirm the role of NLRP3-mediated pyroptosis in the necrosis of the flap, we conducted a NLRP3 inhibition study by using the NLRP3 inhibitor MCC950. Immunofluorescence staining and western blotting exhibited that MCC950 downregulated the expressions of the pyroptosis related proteins (Figures 2A–F). Moreover, the caspase-1 and GSDMD-N fluorescence intensities were reduced by treatment with MCC950 compared to the control group. These results further showed that NLRP3 inflammasome triggers the activation of pyroptosis in the flaps.

On the third day after surgery, the random skin flaps in each group were pale and swollen without obvious necrosis in area III. Survival of the flaps in these two groups was not significantly different (Figure 2G). On the seventh day after surgery, each group exhibited survival in Area I, however, Area III had become dark, and necrosis spreads to area II (Figure 2G). Treatment with MCC950 showed improved flap survival compared with that of the control group (Figure 2H). In addition, the MCC950 group displayed lower edema and subcutaneous venous congestion than the control group (Figure 2I). The tissue water content of the MCC950 group was lower than that of the control group (Figure 2J). Vascular network regeneration was visualized by LDBF (Figure 2K), which the higher blood flow intensity of MCC950 group was higher than control group (Figure 2L). MCC950 treatment also increased the number of microvessels and CD34 positive cells in the flap (Figures 2M–P). Data from histological analysis and western blotting indicated VEGF expressions were much larger in the MCC950 group than the control group (Figures 2Q–T). Western blotting analysis showed that MCC950 upregulated the expression of angiogenesis-related proteins including cadherin-5 (Figures 2S,T). Together, the above data suggested that NLRP3 mediated pyroptosis in the random skin flap and participated in the necrosis of the flap.

### Pyroptosis in Flaps Is Dependent on ROS-Induced Lysosomal Malfunction

**FIGURE 3:**
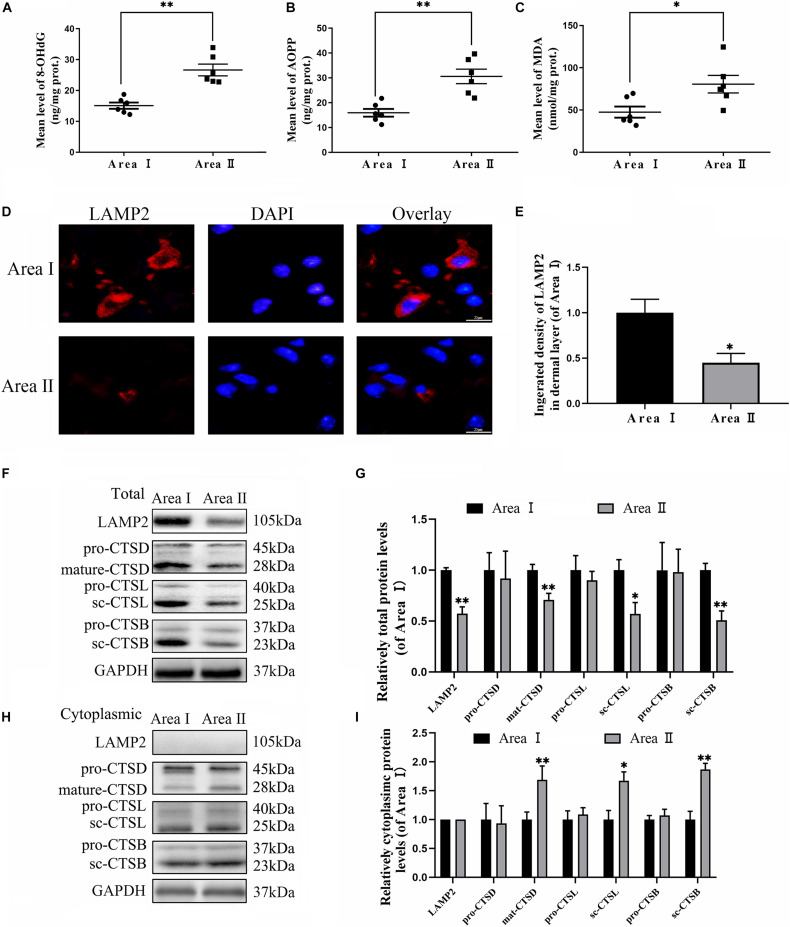
Accumulation of ROS and lysosomal dysfunction exists in random flaps. **(A–C)** ELISA of MDA 8-OHdG and AOPP and in flap tissue from area II and area I. **(D)** Immunofluorescence for LAMP2 in skin flaps (scale bar: 25 mm). **(E)** The fluorescence intensity for LAMP2. **(F,H)** Western blotting of the lysosome function-related proteins CTSD, LAMP2, CTSB, and CTSL in total protein and cytoplasmic protein (isolating lysosomal protein). **(G,I)** Quantification of CTSD, LAMP2, CTSB, and CTSL immunoblots. Values represent the mean ± SEM, *n* = 6 per group. *p < 0.05 and **p < 0.01, vs. area I group.

**FIGURE 4:**
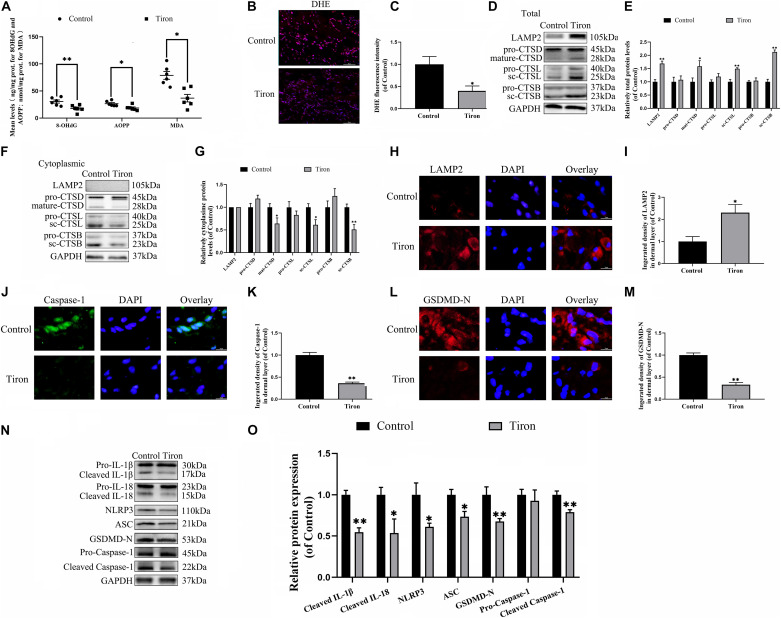
ROS-induced lysosomal malfunction leads to initiation in flaps. **(A)** ELISA of MDA, 8-OHdG, and AOPP and in flap tissue from the control and Trion groups. **(B,C)** DHE staining for sections was conducted to determine the ROS levels (scale bar: 50 mm). **(D,F)** Western blotting of the lysosome function-related proteins CTSD, LAMP2, CTSB, and CTSL in total protein and cytoplasmic protein from the control and Trion groups. **(E,G)** Quantification of CTSD, LAMP2, CTSB, and CTSL immunoblots. **(H)** Immunofluorescence for LAMP2 in flaps (scale bar: 25 mm). **(I)** The fluorescence intensity of immunofluorescence staining of LAMP2. **(J)** Immunofluorescence to evaluate the caspase-1 level (scale bar: 25 mm). **(K)** The fluorescence intensity of caspase-1. **(L)** Image of the immunofluorescence staining of GSDMD-N in skin flaps (scale bar: 25 mm). **(M)** The fluorescence intensity of GSDMD-N analyzed using ImageJ. **(N)** Western blotting of pyroptosis related proteins in the control and Trion groups. **(O)** Quantification of pyroptosis related proteins immunoblots. Values represent the mean ± SEM, *n* = 6 per group. *p < 0.05 and **p < 0.01, vs. control group.

To evaluate the variance in ROS levels after flap surgery, we conducted research on the ROS oxidation productions of MDA, 8-OHdG and AOPP by ELISA. As expected, higher levels of ROS oxidation productions were detected in intermediate flap (area II) compared to the proximal flap (area I) (Figures 3A–C). To evaluate the changes in lysosomal functions after flap operation, western blotting, and immunofluorescence staining were conducted on the tissue of mouse flap area I and area II. The protein expressions of mature-CTSD, LAMP2, sc-CTSL, and sc-CTSB in the total protein were reduced in distal ischemic skin flaps (area II); while mature-CTSD, sc-CTSL, and sc-CTSB in the cytoplasmic protein (isolating lysosomal protein) were increased (Figures 3F–I), which indicated there is tissue lysosome rupture and malfunction in the distal ischemic necrosis of random flaps. The immunofluorescence of LAMP2 also showed the similar result (Figures 3D–E). The above results suggested a potential causal relationship between ROS cumulation and lysosomal dysfunction following flap surgery. Then, our research aimed to verify whether in the distal ischemic necrosis of random flaps, ROS induced lysosomal malfunction and then induced cell pyroptosis. The pre-treatment with Trion, a ROS scavenger, was performed for mice. The results showed that Trion inhibited ROS level and its oxidation productions in the flap (Figures 4A–C), at which time the levels of mature-CTSD, LAMP2, sc-CTSL, and sc-CTSB in the total protein were all increased and mature-CTSD, sc-CTSL, and sc-CTSB in the cytoplasmic protein decreased (Figures 4D–G). Besides, the immunofluorescence of LAMP2 also suggested the similar result (Figures 4H–I). Furthermore, Trion downregulated pyroptosis, as indicated by the decreased expressions of the pyroptosis related markers via immunofluorescence staining and western blotting (Figures 4J–O). These data indicate that pyroptosis was dampened via eliminated ROS-induced lysosomal malfunction in the flap.

Finally, we examined the effect of Trion on flap survival. On day 3, there were no significant disparity in the flap survival area between the control and Tiron groups (Supplementary Figure 1A), higher percentages of flap survival were observed in the Trion group on POD 7 (Supplementary Figures 1A,B). Similarly, the lesser water content in the Tiron group was than the control group (Supplementary Figures 1C,D). Furthermore, Trion groups show higher blow flow intensities than the control group (Supplementary Figures 1E,F). Trion treatment also augmented the number of microvessels and CD34 positive cells in the flap (Supplementary Figures 1G–J). Overall, these sets of experiments indicated that reduced ROS-induced lysosomal malfunction can inhibit pyroptosis as well as promote survival and decrease necrosis in flap tissues.

### TFE3 Ameliorates ROS-Induced Lysosomal Malfunction and Downregulates Pyroptosis in Flaps

**FIGURE 5:**
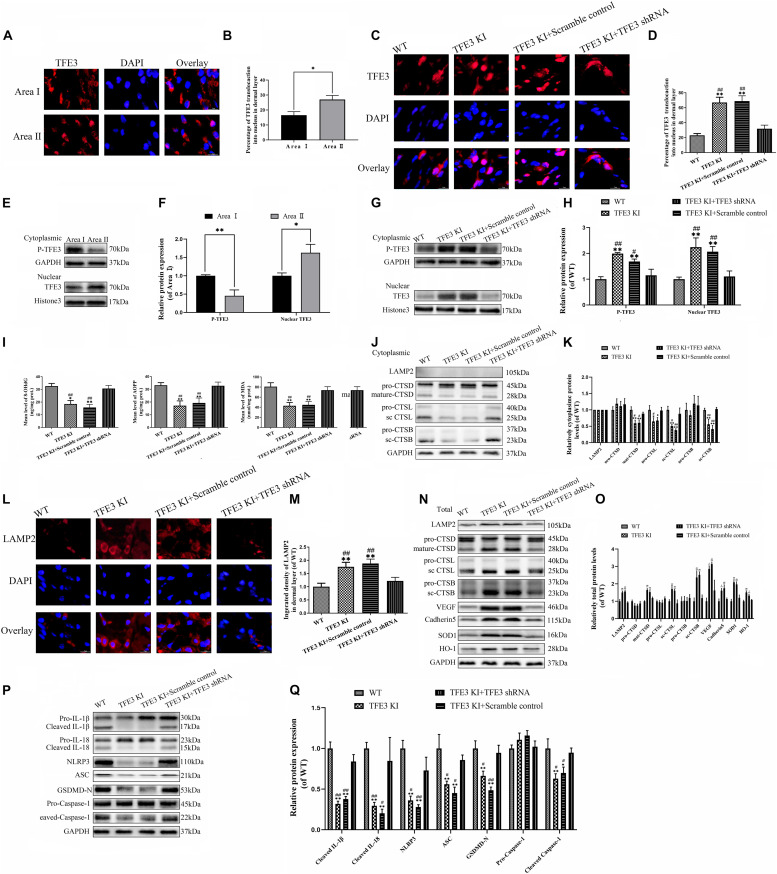
TFE3 ameliorates ROS-induced lysosomal malfunction and downregulates pyroptosis in random skin flaps. **(A,E)** Immunofluorescence exhibiting the nuclear translocation of TFE3 (red) (scale bar: 25 mm). **(B,F)** Percentage of TFE3-positive cells. **(C,G)** Western blot exhibited cytoplasmic TFE3 and nuclear TFE3. **(D,H)** Quantification of cytoplasmic and nuclear TFE3 proteins immunoblots. **(I)** ELISA of 8-OHdG, AOPP, and MDA in flap tissue from WT, TFE3 KI, TFE3 KI + scramble and TFE3 KI + TFE3 shRNA groups. **(J,N)** Western blotting of the lysosome function-related proteins CTSD, LAMP2, CTSB, and CTSL in total protein and cytoplasmic protein. **(K,O)** Densitometric analysis of CTSD, LAMP2, CTSB, and CTSL. **(L)** Immunofluorescence for LAMP2 in skin flaps (scale bar: 25 mm). **(M)** The fluorescence intensity for LAMP2. **(N)** Western blotting of VEGF, cadheirn-5, SOD1, and HO-1 in flap tissue from WT, TFE3 KI, and TFE3 KI + scramble and TFE3 KI + TFE3 shRNA groups. **(O)** Densitometric analysis of VEGF, cadherin-5, SOD1, and HO-1. **(P)** Western blotting of the pyroptosis related proteins. **(Q)** Quantification of pyroptosis related proteins immunoblots. Values are shown as the mean ± SEM, *n* = 6 per group. *p < 0.05 and **p < 0.01, vs. WT group or area I group. ^#^*p* < 0.05 and ^##^*p* < 0.01, vs. TFE3 KI + TFE3 shRNA group.

To evaluate the changes in the TFE3 expressions levels after flap surgery, we conducted a study of the nuclear translocation of TFE3 by immunofluorescence staining and western blotting. Similar to the ROS level and lysosomal dysfunction, higher levels of the nuclear translocation of TFE3 were detected in the distal flap (area II) compared to the proximal flap (area I) (Figures 5A,B,E,F). These findings showed that TFE3 may exert an overriding effect in ROS-induced lysosomal malfunction in flap necrosis. To evaluate the role of TFE3 expression on ROS-induced lysosomal dysfunction and pyroptosis in flap tissues, we used TFE3-KI/wt mice to specifically overexpress TFE3. In addition, a TFE3 shRNA AAV vector was applied in TFE3-KI/wt mice to downregulate TFE3 for further verifying the role of TFE3 expression in ROS-induced lysosomal dysfunction and pyroptosis in the flap. Western blotting results showed that TFE3-KI mice had upregulated TFE3 expression in cell nuclei compared to the WT group, and the AAV-TFE3 shRNA injection downregulated TFE3 expression in cell nuclei compared to the TFE3-KI and TFE3-KI + scramble control groups (Figures 5G,H). Immunofluorescence suggested that TFE3-KI mice were capable of increasing the TFE3 in cell nuclei level, and AAV-TFE3 shRNA treatment was capable of depressing the TFE3 level in random skin flaps (Figures 5C,D). Then, we investigated whether TFE3 can alleviate ROS-induced lysosomal malfunction and then pyroptosis. As shown in Figure 1, we found that the expressions of ROS oxidation productions were downregulated in the TFE3-KI and TFE3-KI + scramble control groups compared with WT mice and TFE3-KI + TFE3 shRNA mice. In addition, we found that the protein levels of anti-oxidation proteins targeted by TFE3, the higher level of SOD1, and HO-1 were detected in the TFE3-KI and TFE3-KI + scramble control groups compared with the WT and TFE3-KI + TFE3 shRNA groups (Figures 5N,O). Western blotting suggested that the levels of the lysosome function-related proteins mature-CTSD, LAMP2, sc-CTSL, and sc-CTSB in the total protein were upregulated in the TFE3-KI and TFE3-KI + scramble control groups compared with the WT and TFE3-KI + TFE3 shRNA groups; while mature-CTSD, sc-CTSL, and sc-CTSB in the cytoplasmic protein were opposite (Figures 5J,K,N,O). Immunofluorescence staining showed the similar results (Figures 5L,M). These data indicated that ROS-induced lysosomal malfunction in flaps can be depressed by the transcriptional overexpression of lysosome biogenesis and anti-oxidation genes targeted by TFE3.

The results from western blotting revealed that the lower expressions of the pyroptosis-related markers were observed in the TFE3-KI and TFE3-KI + scramble control groups compared with the WT and TFE3-KI + TFE3 shRNA groups (Figures 5P,Q). As expected, the TFE3-KI and TFE3-KI + scramble control groups showed higher percentages of viability in the flaps on P0D7 compared with the WT and TFE3-KI + TFE3 shRNA groups (Supplementary Figures 2A,B). In addition, compared with the WT and TFE3-KI + TFE3 shRNA groups, the water contents were decreased in the TFE3-KI and TFE3-KI + scramble control groups (Supplementary Figures 2C,D). LDBF analysis indicated that TFE3-KI mice and TFE3-KI + scramble control mice had better blood flow signal intensities compared with the mice of the WT and TFE3-KI + TFE3 shRNA groups (Supplementary Figures 2E,F). Meanwhile, a higher number of microvessels was observed in the TFE3-KI and TFE3-KI + scramble control groups (Supplementary Figures 2G,H). Finally, western blotting showed that the levels of VEGF and cadherin-5 were higher in the TFE3-KI and TFE3-KI + scramble control groups (Figures 5N,O). Overall, these sets of experiments suggested that TFE3 abates ROS-induced lysosomal malfunction, inhibits pyroptosis, promotes survival and decreases necrosis in flap tissues.

### TFE3 Silencing Aggravates ROS-Induced Lysosomal Malfunction and Promotes Pyroptosis in the OGD/R Cell Model

**FIGURE 6:**
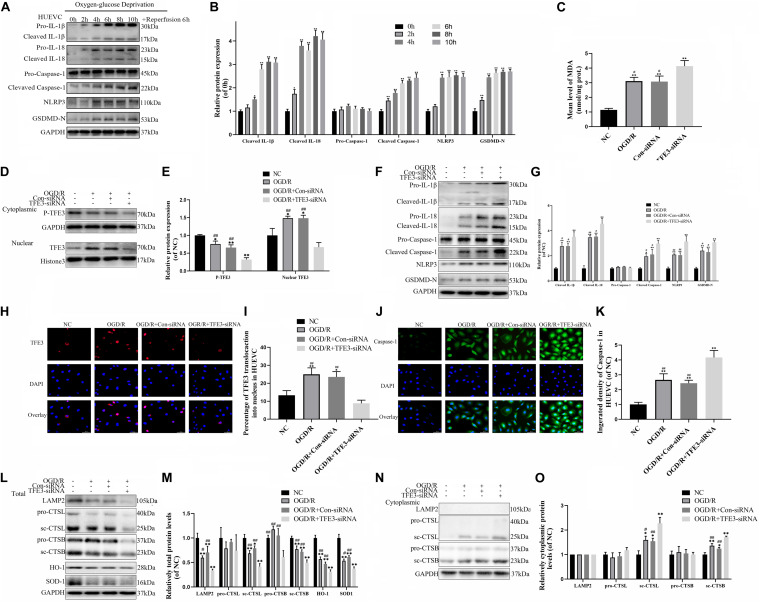
TFE3 silencing aggravates lysosomal malfunction and promotes pyroptosis in the OGD/R cell model. **(A)** Western blotting of the pyroptosis markers in HUVECs. **(B)** Quantification of pyroptosis related proteins immunoblots in HUVECs. **(C)** ELISA of the oxidation product MDA in HUVECs from NC, OGD/R, OGD/R + Con-siRNA, and OGD/R + TFE3-siRNA groups. **(D)** Western blotting of the level of cytoplasmic and nuclear TFE3 in HUVECs. **(E)** Quantification of TFE3 immunoblots. **(F)** The protein levels of pyroptosis related proteins in HUVECs from each group were detected via western blotting. **(G)** Quantification of pyroptosis related proteins. **(H)** Immunofluorescence was performed to exhibit the nuclear translocation of TFE3 (red) in the HUVECs (scale bar: 25 mm). **(I)** Percentage of TFE3-positive cells. **(J)** Immunofluorescence to detect the caspase-1 expression level was conducted to exhibit the pyroptosis level in the HUVECs (scale bar: 25 mm). **(K)** The fluorescence intensity of caspase-1 analyzed using ImageJ. **(L,N)** Western blotting of LAMP2, CTSL, CTSB, SOD1, and HO-1 in HUVECs from each group. **(M,O)** Western blot and its quantification showed the level of LAMP2, CTSL, CTSB, SOD1, and HO-1. Data are the mean ± SEM, *n* = 6 per group. *p < 0.05 and **p < 0.01, vs. NC group. ^#^*p* < 0.05 and ^##^*p* < 0.01, vs. OGD/R + TFE3-siRNA group.

To further verify the relationship between ROS-induced lysosomal malfunction, pyroptosis, and TFE3 in HUVEC cells, we examined whether reduced expression of TFE3 augments ROS-induced lysosomal malfunction and subsequent aggravates pyroptosis in cells *in vitro*. TFE3 silencing was performed using TFE3 siRNA transfection. Similar with vivo research, western blotting and immunofluorescence showed that higher TFE3 nuclear translocation was observed in the OGD/R group compared with the NC group (Figures 6D,E,H,I). TFE3 siRNA downregulated the TFE3 level in cell nuclei relative to the ODG/R and ODG/R + Con-siRNA groups (Figures 6D,E). Immunofluorescence revealed that TFE3 siRNA treatment was indeed able to depress TFE3 expression in HUVECs (Figures 6H,I).

Next, we explored if the reduced expression of TFE3 can trigger aggravation of a ROS-induced lysosomal malfunction and subsequent pyroptosis in cells. As depicted in Figure 6C, and Supplementary Figures 3A,B, the levels of ROS and MDA were lower in the NC compared with the ODG/R and ODG/R + Con-siRNA groups and were higher in the ODG/R + TFE3-siRNA group compared with the ODG/R and ODG/R + Con-siRNA groups. In addition, we found that the protein levels of anti-oxidation proteins, SOD1, and HO-1 were downregulated in the ODG/R + TFE3-siRNA group compared with the ODG/R and ODG/R + Con-siRNA groups and increased in the NC group compared with the ODG/R and ODG/R + Con-siRNA groups (Figures 6L,M). Furthermore, western blotting showed that the levels of lysosome function-related proteins sc-CTSB, LAMP2, and sc-CTSL in total protein were reduced in the ODG/R + TFE3-siRNA group compared with the ODG/R and ODG/R + Con-siRNA groups and were increased in the NC group compared with the ODG/R and ODG/R + Con-siRNA groups, while sc-CTSB and sc-CTSL in the cytoplasmic protein were opposite (Figures 6L,M). Together, the above data suggest that ROS-induced lysosomal malfunction *in vitro* can be directly induced by the transcriptional downregulation of lysosome biogenesis and anti-oxidation genes by TFE3.

Western blotting indicate that the expressions of pyroptosis related markers in the ODG/R + TFE3-siRNA group were increased, and in the NC group, they were decreased compared to the other two groups (Figures 6F,G). The immunofluorescence of caspase-1 showed similar results (Figures 6J,K). As shown in Supplementary Figures 3C,D, under the TFE3-siRNA condition, the cells migrated slower than in the ODG/R and ODG/R + Con-siRNA groups, as calculated with the Transwell assay. At the same time, experiments with the tube formation assay implied that the TFE3-siRNA treatment inhibited the tube formation ability of HUVECs (Supplementary Figures 3E,F). These results implied that TFE3 silencing promotes pyroptosis and affects the migration and tube formation of HUVECs.

### The Activity of TFE3 Is Regulated by the AMPK-MCOLN1-Calcineurin Signaling Pathways

**FIGURE 7:**
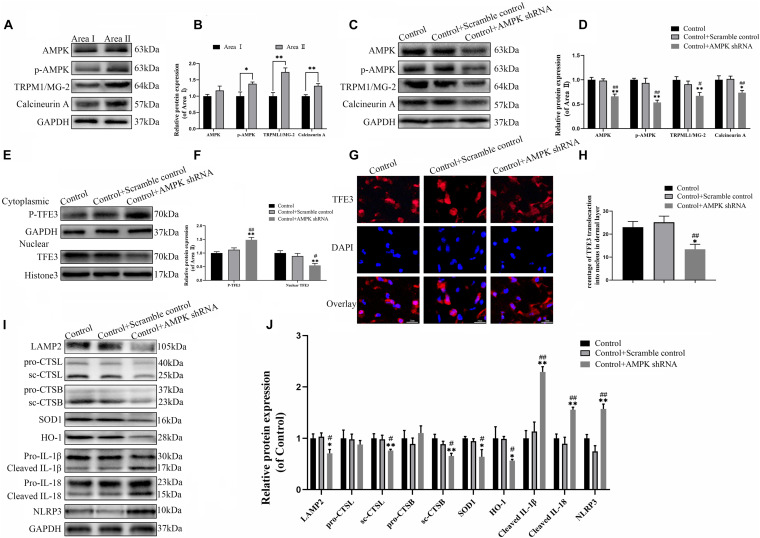
The activity of TFE3 is regulated by AMPK-MCOLN1-calcineurin signaling pathways. **(A,C)** Western blotting of AMPK, p-AMPK, TRPML1/MG-2, and calcineurin in flap tissue. **(B,D)** Quantification of AMPK, p-AMPK, TRPML1/MG-2, and calcineurin immunoblots. **(E)** Western blotting of cytoplasmic TFE3 and nuclear TFE3 in flaps. **(F)** Quantification of cytoplasmic TFE3 and nuclear TFE3 immunoblots. **(G)** Immunofluorescence of nuclear translocation of TFE3 in flaps (scale bar: 25 mm). **(H)** Percentage of TFE3-positive cells. **(I)** Western blotting of LAMP2, CTSL, CTSB, SOD1, HO-1, cleaved-IL-1β, cleaved-IL-18, and NLRP3. **(J)** Histograms of the optical density values of LAMP2, CTSL, CTSB, HO-1, SOD1, cleaved-IL-1β, cleaved-IL-18, and NLRP3. Data are the mean ± SEM, *n* = 6 per group. *p < 0.05 and **p < 0.01, vs. control or area II group. ^#^*p* < 0.05 and ^##^*p* < 0.01, vs. control + scramble control group.

The last question to be clarified is how the activity of TFE3 is regulated by the signaling pathway in the distal ischemic necrosis of flaps. Here, AMPK, as a decisive responder to hunger and low energy states, has been proven to be a crucial regulator of MiTF/TFE family activities, which has attracted widespread attention. MiTF/TFE family nuclear translocation is closely related to the activation of the AMPK- MCOLN1 signaling pathway in the cytoplasm. The western blotting data showed that the expression of phosphorylated AMPK, MCOLN1 and calcineurin in the distal flap (area II) was increased relative to the proximal flap (area I), while the ratios of p-AMPK/AMPK were unchanged (Figures 7A,B). It has been reported that the increase of AMPK activates the MCOLN1 channel, the only lysosomal Ca2 + channel, which subsequently activates PPP3/calcineurin and TFEB-nuclear translocation, finally upregulating the expression of a large series of genes which encodes lysosomal proteins (lysosomal biogenesis) and leads to the normalization of lysosomal function and mitigation of ROS (Zhang et al., 2016; Fernandez-Mosquera et al., 2019). TFE3 and TFEB often share regulatory signaling networks, and thus, we hypothesize that the activity of TFE3 in the nucleus in the ischemic flap may be regulated by the AMPK-MCOLN1 pathway.

To confirm the relationship between the TFE3 activity and AMPK-MCOLN1 signaling pathways in flaps, an AMPK shRNA AAV vector was applied to knock-down AMPK. The data from western blotting revealed that AAV-AMPK shRNA injection downregulated the AMPK-MCOLN1-calcineurin pathways in random skin flaps and decreased the nuclear translocation of TFE3 compared with the control and control + scramble control groups (Figures 7C–F). Meanwhile, the immunofluorescence showed that the nuclear translocation of TFE3 was lower in the AAV-AMPK shRNA group (Figures 7G,H). In summary, these findings suggested that the activity of TFE3 in flaps is partly regulated via the AMPK-MCOLN1-calcineurin signaling pathways. Anti-oxidation protein expression and lysosome protein function were evaluated as well, and as shown in Figures 7I,J, SOD1, HO-1, sc-CTSL, LAMP2, and sc-CTSB, expression levels were lower in the AAV-AMPK shRNA group when compared to the other two groups. In addition, AAV-AMPK shRNA treatment promoted the expression of pyroptosis-related protein (Figures 7I,J). These results indicate that AAV-AMPK shRNA injection treatment augments ROS-induced lysosomal malfunction and aggravates pyroptosis in random flaps.

## Discussion

This study first verified the role of pyroptosis in random skin flaps and further provides new evidence that TFE3 is a promising regulator of ROS-induced pyroptosis in random flaps and HUVECs. We found that TFE3 directly affects the level of ROS in the flap, and ROS induced by an ischemic flap initiates lysosomal dysfunction, subsequently triggering cell pyroptosis. In addition, we revealed that the AMPK-MCOLN1 signaling pathway may exert a decisive activation effect on TFE3 in ischemic flaps. Finally, and most importantly, we found that the regulation of TFE3 levels leads to profound consequences for the necrosis of flaps in the mice model, indicating that TFE3 may be a potential target for ischemic flap therapy.

Pyroptosis is also known as gasdermin-mediated programmed necrosis (Shi et al., 2017), which was first found in Salmonella-induced macrophage death (Brennan and Cookson, 2000). In addition, pyroptosis is also characterized by pore formation in the cell membrane, cell swelling and membrane rupture (He et al., 2015; Ge et al., 2018). Different from apoptosis, pyroptosis can lead to the release of inflammatory factors, which activate pro-inflammatory immune mediators and then trigger a series of amplified inflammatory responses (Wu et al., 2019). Recently, multiple signaling pathways have been found to mediate the occurrence of pyroptosis, among which, NLRP3-caspase-1-GSDMD is the most classical (He et al., 2015). The typical pyroptosis mediated by caspase-1 can be activated by seven inflammasomes including NLRP3, NLRP1, NLRP6, NLRP9, AIM2, NLRC4, and pyrin (Heilig and Broz, 2018). Among them, NLRP3 is considered to be an important NOD-like receptor protein that can recognize a variety of pathogen-associated molecular patterns (PAMPs) and danger-associated molecular patterns (DAMPs) and initiate a sterile inflammatory response (Mangan et al., 2018; Wang S. et al., 2019). It activates caspase-1 by transduction of recognition signals to the inflammasome adaptor ASC (apoptosis-related speck-like protein). Subsequently, the activated caspase-1 cleavage and/or maturation of pro-IL-1β, pro-IL-18, and Gasdermin D causes macropores in the cell plasma membrane, leading to cell pyroptosis (Pétrilli et al., 2007; Hosseinian et al., 2015; Wang Y. et al., 2019). Pyroptosis has been found in numerous organs and tissues with IR injury (Li et al., 2018). Besides, pyroptosis exerts overriding effects in cardiovascular diseases (Wan et al., 2020), such as atherosclerosis (Wu X. et al., 2018), diabetic cardiomyopathy (Li et al., 2014), and myocardial ischemia/reperfusion injury (Nazir et al., 2017). As a vascular disease, the necrosis of random flaps is not only featured by microvascular disease, but also by tissue ischemia-reperfusion injury (Mao et al., 2019). Thus, NLRP3-mediated pyroptosis may also exert a decisive role in the ischemic necrosis of random flaps. However, in the area of ischemic flaps, there has been no research on pyroptosis and its specific regulation mechanism. In the current study, we found that the NLRP3-mediated pyroptosis expressions were activated both in flap animal model and in the OGD/R cell model. This result implies that NLRP3-pyroptosis may exists in the necrosis of ischemic flaps. Many researchers have shown that NLRP3-mediated pyroptosis leads to the occurrence and development of many diseases (Zhou et al., 2018; Al Mamun et al., 2020). Here, we hypothesized that NLRP3-mediated pyroptosis leads to the necrosis of flaps. To test our hypothesis, MCC950, the inhibitor of NLRP3, was used in current research, showing that the inhibitor downregulated the level of pyroptosis-related proteins and promoted the survival area of the random flaps. Together, the above data supported that NLRP3-mediated pyroptosis leads to the necrosis of flaps.

Pyroptosis is often accompanied by the production of ROS, and damage to organelles, such as lysosomal dysfunction, which are considered to be important causes of pyroptosis (Pétrilli et al., 2007). Specifically, with lysosomal malfunction, increased membrane permeability triggers the activation and release of cathepsin B into the cytoplasm, followed by binding and activating NLRP3 to initiate pyroptosis (Jia et al., 2019). ROS destroy the stability of lysosomes through oxidizing unsaturated fatty acids in the lysosomal membrane to permeate the membrane and then inducing the extravasation of proteolytic enzymes (Pétrilli et al., 2007; Chen et al., 2012; Liu et al., 2013). Additionally, ROS upregulated the NF-κB signaling axis, which activated NLRP3-inflammasome and finally triggered pyroptosis (Liu et al., 2013; Jia et al., 2019). Extensive studies have demonstrated that ischemia reperfusion injury causing ROS accumulation in functional cells is the main factor leading to the necrosis of random skin flaps (Suzuki et al., 1989). Therefore, we speculated that ROS induce lysosome malfunction and subsequently trigger pyroptosis in ischemic flaps. In current research, the overproduction of ROS was detected in the distal flap, which coincided with lysosome dysfunction in the distal flap. For *in vitro* research, the ROS level of the cells increased, and the lysosome activity decreased after OGD/R treatment. The above *in vivo* and *in vitro* results suggested a potential causal relationship between ROS accumulation with lysosomal malfunction following flap surgery. Furthermore, we also found that the Tiron (a ROS scavenger) inhibited ROS-induced lysosomal malfunction and pyroptosis and enhanced the viability of the random flaps, suggesting that ROS accumulation can induce lysosome malfunction, leading to the pyroptosis in random flaps later.

The MiT/TFE family, involving MITF, TFEB, TFE3, and TFEC, exert a decisive effect in regulating lysosomal function and oxidative metabolism, which has attracted a lot of attention (Sardiello et al., 2009; Wu B. et al., 2018). Under cellular stress, MITF, TFEB, and TFE3 all trigger the expression of a set of transparent elements on lysosomal genes (such as CTDS and LAMP2), thus enhancing the biogenetic activity of lysosomes and promoting the biogenetic activity of lysosomes (Zhou et al., 2013; Asrani et al., 2019; Ozturk et al., 2019). Moreover, TFEB and TFE3 can downregulate intracellular ROS by promoting the expression of antioxidant genes such as HO1, and Sod (Martina and Puertollano, 2016; Lu et al., 2017). Consequently, these transcription factors may underlie the pathophysiology of necrosis of flaps and thus may be applied for therapeutic benefits. Our findings showed that higher levels of the nuclear translocation of TFE3 were detected in the distal flap (area II) compared to the proximal flap (area I), which coincided with ROS accumulation and lysosomal malfunction. Hence, we speculated that TFE3 dampens the level of oxidative stress and promotes lysosomal function and biogenesis to inhibit cell pyroptosis, finally enhancing the survival of the distal end of random flaps. To test the hypothesis, we used TFE3 knock-in transgenic mice to over-express TFE3, showing that increased TFE3 inhibited ROS-induced lysosome dysfunction and subsequent cell pyroptosis, ultimately promoted the viability of random skin flaps. AAV-TFE3 shRNA was used to downregulate TFE3 expression in TFE3 knock-in transgenic mice. We found that the effect of over-expression TFE3 in ROS-induced lysosome dysfunction, cell pyroptosis, and the viability of random skin flaps were reversed by AAV-TFE3 shRNA. The above conclusion suggested that TFE3 depressed pyroptosis via alleviating ROS accumulation and ROS-induced lysosomal malfunction in animal models.

To further verify the effect of TFE3 in the OGD/R cell model consistent with the results of animal experiments, we used TFE3 siRNA transfection to silence TFE3. First, after OGD/R treatment, TFE3 was activated, ROS and pyroptosis levels of the cells increased, and the lysosome activity decreased, accompanied by reduced cellular activity for *in vitro* research. Then, our results demonstrated that TFE3 silencing aggravates ROS-induced lysosomal malfunction, promotes pyroptosis and finally inhibits cell viability in the OGD/R cell model. It can be concluded that TFE3 promotes HUVEC viability by inhibiting ROS and ROS-induced lysosome dysfunction, thereby reducing pyroptosis. TFEB, another member of MiTF/TFE family, also dampen ROS by promoting the expression of antioxidant genes (Wu B. et al., 2018; Ozturk et al., 2019). Therefore, TFEB may also be a potential target in inhibiting pyroptosis via the above mechanism, which needs to be explored in the future research.

Finally, we explored the TFE3 specific upstream regulation mechanism. AMPK, as an important reactant to hunger and low energy states, has been as a crucial regulator of MiTF/TFE family activities, which has attracted widespread attention (Slade and Pulinilkunnil, 2017). Nuclear translocation in MiTF/TFE family are closely related to the activation of the AMPK- MCOLN1 signaling pathway in the cytoplasm (Zhang et al., 2016; Fernandez-Mosquera et al., 2019). Increased AMPK has been found to activate the MCOLN1 channel, which subsequently induces lysosomal Ca^2+^ release, leading to PPP3/calcineurin activation (Medina and Ballabio, 2015; Fernandez-Mosquera et al., 2019). Calcineurin activation decreases the rate of TFE3/TFEB phosphorylation and induces TFE3/TFEB dephosphorylation, which finally promotes TFE3/TFEB nuclear translocation and induces the expression of a unique set of genes (Roczniak-Ferguson et al., 2012; Settembre et al., 2012; Yin et al., 2017; Zhao and Zhang, 2019). We found that the AMPK-MCOLN1-calcineurin signaling pathway was activated in the distal flap. Futhermore, AAV-AMPK shRNA was revealed to inhibit AMPK-MCOLN1-calcineurin signaling, nuclear translocation of TFE3, and increase ROS-induced lysosomal malfunction and pyroptosis in flaps, indicating that the activity of TFE3 in flaps is partly regulated via AMPK-MCOLN1signaling pathway. As mentioned above, TFEB has been verified to be regulated by AMPK-MCOLN1signaling pathway, but whether MITF can be activated by the signaling pathway is unknown. Considering the shared regulatory network of TFE3, TFEB, and MITF, we deduced that TFEB and MITF could be regulated in ischemic flaps through AMPK-MCOLN1signaling pathway, which needs to be verified in the future research.

Naturally, there are still some limitations in our work that need to be further explored. Recent studies have found that microRNA (miRNA) and long non-coding RNAs (lncRNAs) can regulate TFE3 levels. Therefore, the TFE3 activity may also be regulated by miRNA or lncRNAs. Although current research mainly focuses on pyroptosis, other types of programed cell death, such as necroptosis, and ferroptosis, may be involved in flap necrosis, which may be affected by ROS and lysosome mechanisms. Hence, the results of this research on TFE3, ROS and lysosome are also applicable to the ferroptosis and necroptosis of flaps, which need to be further explored. In addition to lysosome malfunction, pyroptosis can be produced by potassium ion movement. It is possible that pyroptosis in random flaps may also be regulated by potassium ion movement. In our previous study, we found that calcitriol significant promoted survival of random skin flaps (Chen et al., 2017). Calcitriol may has the bio-activity of anti-pyroptosis in the flaps, which is necessary to be verified in the future.

## Conclusion

**FIGURE 8:**
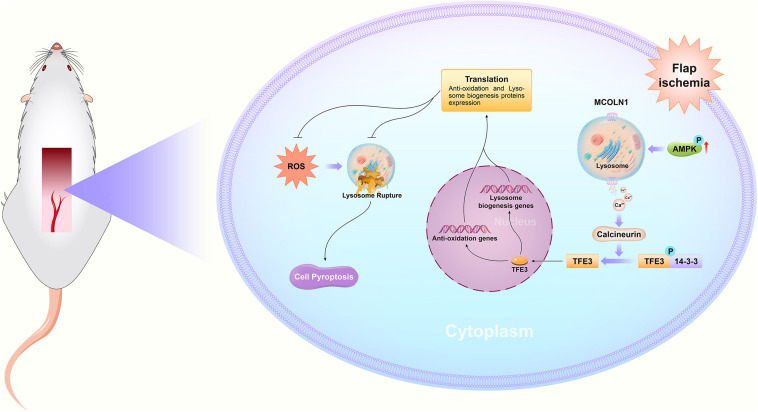
Schematic diagrams emphasizing the pathophysiological molecular mechanisms of TFE3, ROS, and pyroptosis in the necrosis of skin flaps.

In summary, we found that ischemic flaps result in the excessive accumulation of ROS, leading to lysosomal dysfunction and subsequently inducing cell pyroptosis, which ultimately leads to flap necrosis. In addition, we found that in ischemic flaps and OGD/R-induced HUVECs, TFE3 plays an overriding effect in regulating these processes (Figure 8), and it may be a potential therapeutic target for promoting skin flap survival.

## Data Availability Statement

The original contributions presented in the study are included in the article/Supplementary Material, further inquiries can be directed to the corresponding author/s.

## Ethics Statement

The animal study was reviewed and approved by the Wenzhou Medical University’s Animal Research Committee (wydw 2017-096).

## Author Contributions

JiL: writing and review. JuL and RC: data curation. GY and ZC: investigation. CW, JD, and YX: investigation and data curation. HX and XZ: supervision. XZ, WG, and KZ: formal analysis, review, and editing. All authors contributed to the article and approved the submitted version.

## Conflict of Interest

The authors declare that the research was conducted in the absence of any commercial or financial relationships that could be construed as a potential conflict of interest.
