# Supplementary figures and images for: Targeting TFE3 Protects Against Lysosomal Malfunction-Induced Pyroptosis in Random Skin Flaps via ROS Elimination
Source: Front Cell Dev Biol. 2021 Apr 8;9:643996. doi: 10.3389/fcell.2021.643996 (PMC8060706; doi:10.3389/fcell.2021.643996)

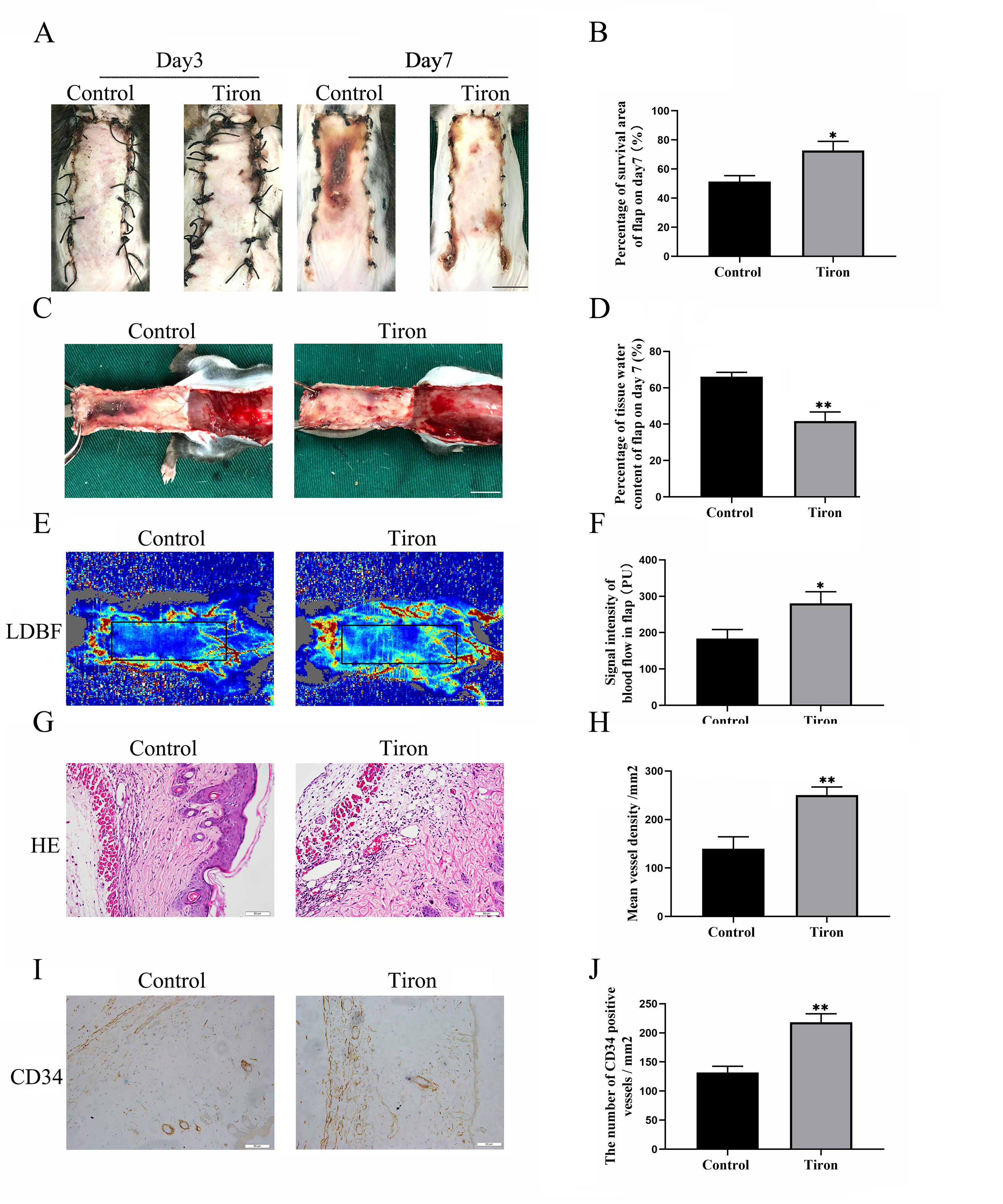

Supplement: Supplementary file 1 [file Image_1.tif]

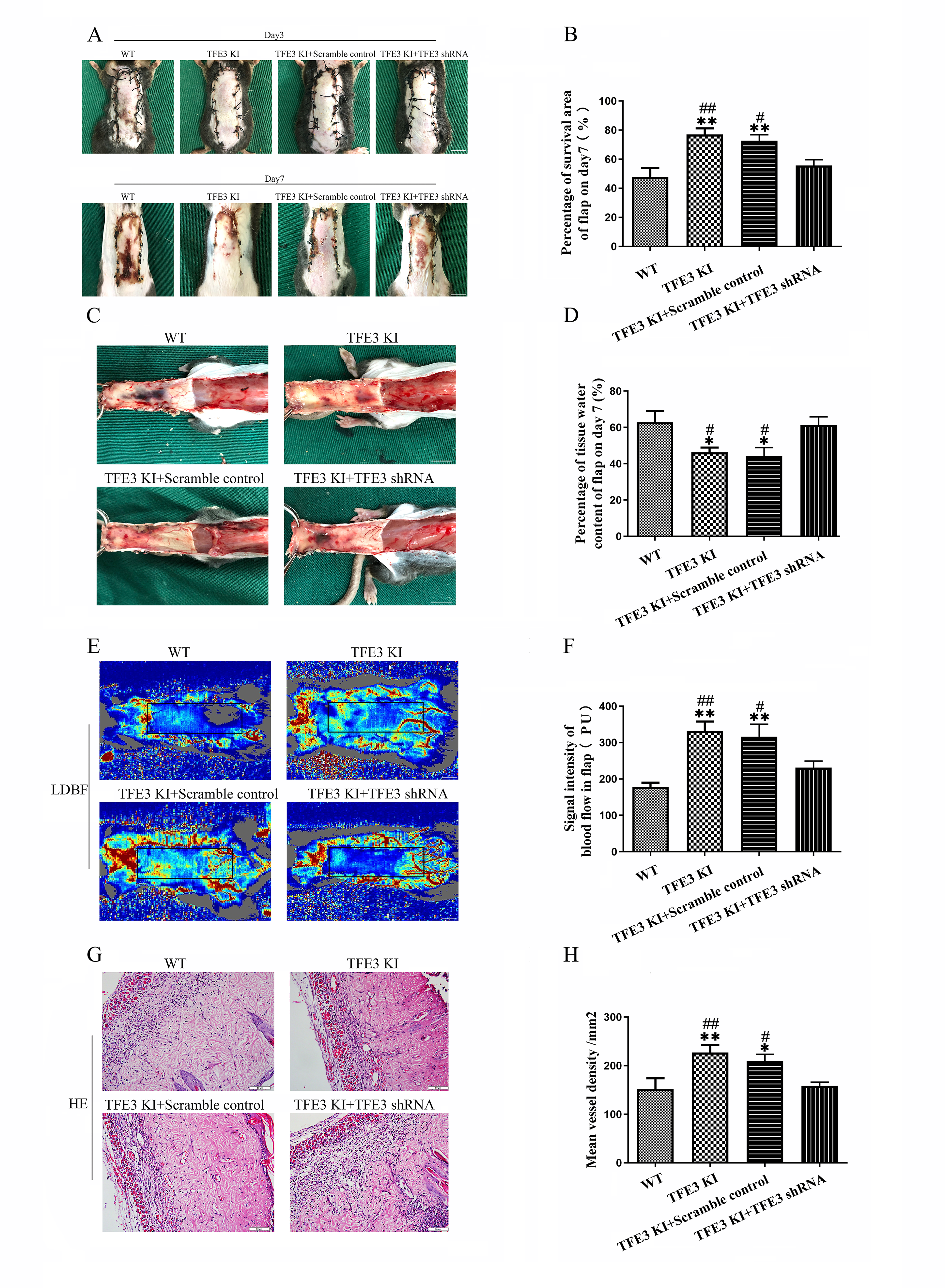

Supplement: Supplementary file 2 [file Image_2.tif]

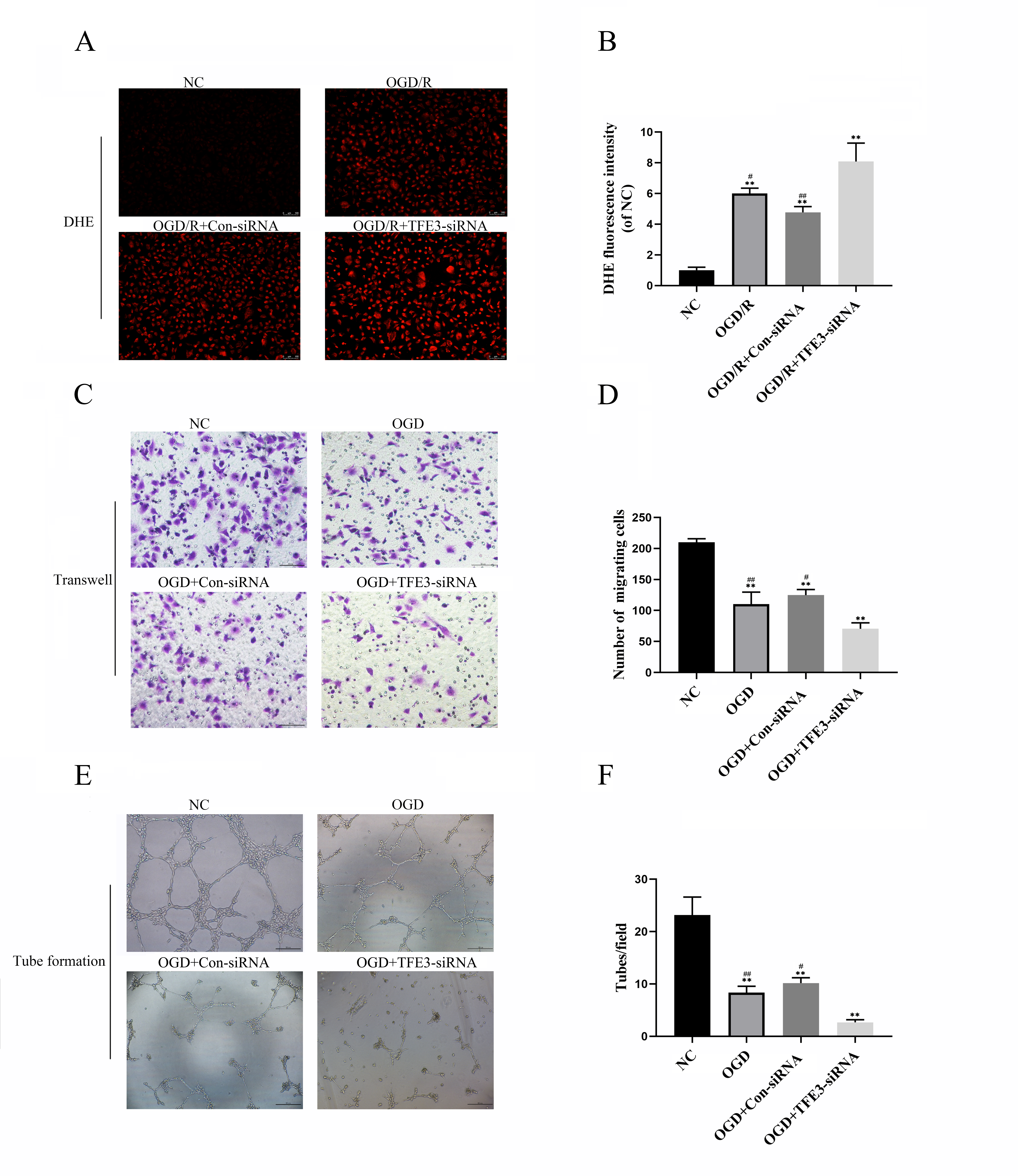

Supplement: Supplementary file 3 [file Image_3.TIF]
