# Supplementary material for: Targeting TFE3 Protects Against Lysosomal Malfunction-Induced Pyroptosis in Random Skin Flaps via ROS Elimination
Source: Front Cell Dev Biol. 2021 Apr 8;9:643996. doi: 10.3389/fcell.2021.643996 (PMC8060706; doi:10.3389/fcell.2021.643996)
Supplement: Supplementary file 4 [file Presentation_1.PDF]

Supplemental Data

Figure S1. ROS elimination promotes the survival of random skin flaps. **(A)** Images of flap necrosis on POD 3 and POD 7 (scale bar, 1.0 cm). **(B)** Histogram exhibiting the percentage of viable flap area. **(C)** Digital images of the inner side of flap. (scale bar, 1.0 cm). **(D)** The percentage of tissue water content was quantified and analyzed. **(E)** Full field LDBF images of flaps (scale bar, 1.0 cm). **(F)** Quantification of signal intensity of blood flow. **(G)** H&E staining showing microvessels in flaps (original magnification, ×200; scale bar, 50 μm). **(H)** Histogram exhibiting the MVD in flaps. **(I)** The expression of CD34 to mark vessels was evaluated by immunohistochemistry staining (Scale bar, 50 μm). **(J)** Histogram showing the CD34-positive vessel densities. Values represent the mean ± SEM, n = 6 per group. $*p < 0.05$ and $**p < 0.01$, vs. control group.

Figure S2. Targeting TFE3 enhances the viability of random skin flaps. **(A)** Images of flap necrosis from each group (scale bar, 1.0 cm). **(B)** Histogram exhibiting the percentage of viable flap area. **(C)** Digital images of the inner side of flap (scale bar, 1.0 cm). **(D)** Quantification of the flap tissue water content. **(E)** LDBF imaging (scale bar, 1.0 cm). **(F)** Quantification of signal intensity of blood flow. **(G)** H&E staining to show vessels (original magnification, ×200; scale bar, 50 μm). **(H)** Histogram exhibiting the MVD in flaps. Values are shown as the mean ± SEM, n = 6 per group. $*p < 0.05$ and $**p < 0.01$, vs. WT group or area I group. $^{\#}p < 0.05$ and $^{\#\#}p < 0.01$, vs. TFE3 KI + TFE3 shRNA group.

Figure S3. TFE3 silencing aggravates ROS and inhibits angiogenesis in the OGD/R cell model. **(A, B)** ROS levels in different groups were measured by the DHE kit under fluorescence microscopy and quantified (scale bar: 100 μm). **(C, D)** The migration of HUVECs from each group was determined by the application of the Transwell assay. **(E, F)** The tube formation of HUVECs was analyzed, and its

quantitative analysis. Data are the mean ±SEM, n = 6 per group. $*p < 0.05$ and $**p < 0.01$, vs. NC group. $^{\#}p < 0.05$ and $^{\#\#}p < 0.01$, vs. OGD/R + TFE3-siRNA group.
